# Patient‐related Predictors for the Functional Outcome of SuperPATH Hemiarthroplasty versus Conventional Approach Hemiarthroplasty: A Systematic Review and Meta‐regression Analysis of Randomized Controlled Trials

**DOI:** 10.1111/os.14006

**Published:** 2024-02-01

**Authors:** Nikolai Ramadanov, Maximilian Voss, Robert Hable, Robert Prill, Hassan Tarek Hakam, Mikhail Salzmann, Dobromir Dimitrov, Roland Becker

**Affiliations:** ^1^ Center of Orthopedics and Traumatology University Hospital Brandenburg an der Havel, Brandenburg Medical School Theodor Fontane Brandenburg an der Havel Germany; ^2^ Faculty of Health Science Brandenburg Brandenburg Medical School Theodor Fontane Brandenburg an der Havel Germany; ^3^ Faculty of Applied Computer Science Deggendorf Institute of Technology Deggendorf Germany; ^4^ Department of Surgical Propedeutics, Faculty of Medicine Medical University of Pleven Pleven Bulgaria

**Keywords:** conventional approaches, hemiarthroplasty, meta‐analysis, meta‐regression, SuperPATH

## Abstract

Specialist literature lacks evidence that explores associations between patient characteristics and the beneficial treatment effect of SuperPATH hemiarthroplasty (HA) compared with conventional approach (CA) HA. To investigate and identify patient‐related predictors of the effect size of the short‐term functional outcome of SuperPATH HA and CA HA by performing a systematic review and meta‐regression analysis of randomized controlled trials (RCTs). A systematic search of literature was performed in PubMed, CNKI, CENTRAL of The Cochrane Library, Clinical trials, and Google Scholar until August 25, 2023. For the continuous outcome parameter Harris hip score (HHS) ≤1 week and 3 months postoperatively, mean differences (MDs) with 95% confidence intervals (CIs) were calculated. A meta‐regression analysis was based on random‐effects meta‐analysis using the Hartung–Knapp–Sidik–Jonkman method for continuous covariates. A total of five RCTs with 404 patients were found. The following predictors of HHS ≤1 week postoperatively were identified: patient age (predictor estimate = 1.29; *p* < 0.01), patient age groups (predictor estimate = 14.07; *p* < 0.01), time to mobilization (predictor estimate = 5.51; *p* < 0.01). The following predictors of HHS 3 months postoperatively were identified: incision length (predictor estimate = −2.12; *p* < 0.01); intraoperative blood loss (predictor estimate = 0.02; *p* < 0.01). Patient age, time to mobilization, incision length, and intraoperative blood loss were identified as predictors of the effect size of early postoperative functional outcome as measured by HHS. Elderly patients, particularly those over 70 years of age, appear to benefit from SuperPATH HA. Based on these findings, and taking into account our limitations, we recommend that the use of minimally invasive SuperPATH HA in elderly patients should be more widely considered and not limited to elective THA patients.

## Graphical abstract

This systematic review and meta‐regression analysis of five randomized controlled trials involving 404 patients found that patient age, time to mobilization, incision length, and intraoperative blood loss are predictors of the effect size of short‐term postoperative functional outcome as measured by Harris hip score. Elderly patients, particularly those over 70 years of age, appear to benefit from SuperPATH hemiarthroplasty. We recommend that the use of minimally invasive SuperPATH hemiarthroplasty in elderly patients should be more widely considered and not limited to elective total hip arthroplasty patients.

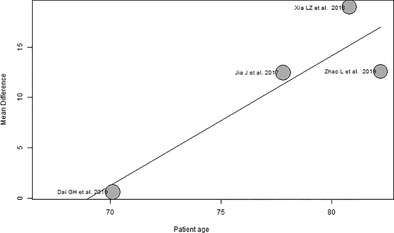

## Introduction

In recent decades there has been steady progress in improving the short‐term postoperative outcomes of patients with elective total hip arthroplasty (THA). This has been achieved through the introduction of minimally invasive approaches and novel techniques. Among these, most notable are the AMIS technique through a direct anterior approach (DAA) approach,^1^ the SuperPATH technique through a direct superior approach (DSA)^2^, ^3^ and the ALMIS technique through an anterolateral approach.^4^ The THA outcome improvements in orthopedic surgery are now being extended to patients with femoral neck fractures undergoing hemiarthroplasty (HA). The latest recommendations are increasingly leaning towards femoral head replacement fracture care, especially in elderly patients, since head‐preserving fracture care leads to high osteosynthesis failure rates and reoperation rates.^5^, ^6^, ^7^ Several meta‐analyses have already shown that patients with femoral neck fractures benefit from minimally invasive surgery using DAA.^8^, ^9^, ^10^, ^11^ Recently, the first meta‐analysis comparing the short‐term outcome of SuperPATH HA and CA HA was published.^12^ This meta‐analysis including nine RCTs and 762 patients showed that SuperPATH leads to a better early functional outcome and decreased incision length, intraoperative blood loss, postoperative drainage volume, time to mobilization, early postoperative pain score, and hospitalization time.^12^ A recent meta‐regression analysis of 41 RCTs with 3607 patients showed that elderly THA patients in particular benefit from minimally invasive surgery.^13^ However, the application of minimally invasive surgery in patients with femoral neck fracture still appears to be underused. The persisting skepticism of traumatologists towards minimally invasive orthopedic techniques and the lower outcome demand of acute traumatological patients than elective orthopedic patients might be regarded as a possible explanation.

Nevertheless, with the publication of the first meta‐analysis of SuperPATH HA versus CA HA and the previously published meta‐analyses of DAA HA versus CA HA, the current study landscape clearly shows an advantage for minimally invasive surgery also in patients with femoral neck fractures.^8^, ^9^, ^10^, ^11^, ^12^ However, the patient cohort of these meta‐analyses is not a homogeneous group and there are certainly many factors that have influenced the results. In an inhomogeneous patient cohort with many influencing factors, it is likely that not all patients will benefit equally from minimally invasive surgery. In this context, there is a need in specialist literature to explore associations between patient characteristics and the beneficial treatment effect of SuperPATH HA compared with CA HA.

The aims of this study were: (i) to systematically review the literature for randomized controlled trials (RCTs) comparing the short‐term functional outcome of SuperPATH HA with CA HA in patients with femoral neck fractures; (ii) to identify patient‐related predictors of the effect size of the short‐term functional outcome of SuperPATH HA and CA HA using meta‐regression analysis; and (iii) to use the results to identify patients who would benefit from either SuperPATH HA or CA HA.

## Methods

### Systematic Review

The present meta‐regression analysis uses the same search strategy as the recently published first meta‐analysis on SuperPATH HA versus CA HA^12^ in patients with femoral neck fractures. This is because the present meta‐regression analysis answers the question of which factors influence the observed treatment effect of the functional outcome from the recently published meta‐analysis.^12^ The present meta‐regression analysis therefore refers to the same study protocol as the recently published first meta‐analysis on SuperPATH HA versus CA HA,^12^ registered in PROSPERO on January 15, 2023 [CRD42023389353], available online at: https://www.crd.york.ac.uk/prospero/display_record.php?RecordID=389353. Similarities between the two studies and with previously published meta‐analyses of this group of authors are due to the use of identical, high‐quality methods.

A systematic search of literature was performed in PubMed, CNKI, CENTRAL of The Cochrane Library, Clinical trials, and Google Scholar until August 25, 2023, using a BOOLEAN search strategy adapted to the syntax of the databases used: ([SuperPATH] OR [supercapsular percutaneously assisted total hip]). Additional trials and gray literature was searched in Google Scholar. The reference lists of retrieved articles and similar meta‐analyses were checked for relevant records. The inclusion criteria were limited to: (i) randomized controlled trials (RCTs); (ii) with human participants (without demographic limitations such as patient age, sex, body mass index [BMI], etc.) with a femoral neck fracture; (iii) operated on with a bipolar HA; and (iv) measuring the functional outcome: Harris hip score (HHS).^14^

### Data Extraction

The following data extraction was performed by two independent reviewers: details of the RCTs (e.g., study design, number of patients, number of operated hips, year of publication, study origin, and risk of bias), primary patient characteristics (e.g., age, sex, BMI), intraoperative patient characteristics, intervention (e.g., approach, cement usage, table position, operation time, incision length, intraoperative blood loss, serum biomarkers), postoperative patient characteristics (e.g., complications), functional outcome (e.g., postoperative HHS). After extracting data on patient age, patients were additionally divided according to their age into an older (≤70) and younger (≥71) patient age group, with an age limit of 71 years being arbitrarily selected. In cases of missing data, a letter requesting additional information was sent to the corresponding authors. If the information on standard deviation was missing, it was calculated by imputation.^15^

### Functional Outcome

The meta‐regression analysis was limited to the most important HA outcome parameter: the functional outcome parameter HHS, measured ≤1 week and 3 months postoperatively. This score was invented to assess the functional outcome of hip surgery^14^ and is based on an assessment of four aspects: pain, function, degree of deformity, and range of motion of the hip. The higher the total score, the better the outcome, with total scores ranging from 0 to 100.

### RCT Quality Assessment

Risk of bias and level of evidence assessment were performed following the Cochrane's risk of bias 2 (RoB 2) tool^16^ and the recommendations of the GRADE system.^17^ In addition, the publication bias^18^ of the RCTs included was assessed, by using the Egger's regression intercept test for asymmetry of the funnel plots (*p*‐value < 0.05). The results in funnel plots to find evidence of publication bias were presented. In the funnel plot, the horizontal axis (*x*‐axis) shows the estimated effect size of the RCTs, and the *y*‐axis shows the estimated standard error of the RCTs (= measure of the uncertainty of the estimated effect size). The dotted vertical line is the overall effect estimated from the meta‐analysis of all RCTs. Ideally, the RCTs should be distributed symmetrically within the triangle.

### Data Synthesis and Statistical Analysis

A professional statistician (RH) performed all statistical calculations using the R packages meta and metafor.^19^ Forest plots show the measures of treatment effect between the SuperPATH HA group and the CA HA group. For the continuous outcome parameter HHS ≤1 week and 3 months postoperatively, mean differences (MDs) with 95% confidence intervals (CIs) were calculated. In the forest plots, a positive MD favors the SuperPATH HA group. Meta‐regression analysis was based on random‐effects meta‐analysis using the Hartung–Knapp–Sidik–Jonkman method for continuous covariates.^20^ Individual covariate regression models were fitted, and heterogeneity was calculated using Cochrane's Q_E test (*p*‐value < 0.10 indicates heterogeneity). The effect of covariates was calculated using the Q_MWald‐type test of model coefficients.

The results of the meta‐regression analysis are presented in bubble plots. The value of the predictor for each RCT is plotted on the horizontal axis (*x*‐axis) and the value of the effect size is plotted on the vertical axis (*y*‐axis). Each RCT is represented by a bubble. The weight of the RCT's contribution to the overall result correlates with the size of the bubbles. The black solid line is the regression line. The slope of the regression line corresponds exactly to the effect size of the predictor on the outcome variable. The slope of the regression line is large (steep line) when the predictor has a large effect on the outcome variable. The regression line will be flat and almost parallel to the horizontal zero line when the predictor has no effect on the outcome variable.

## Results

### Systematic Review

**FIGURE 1:**
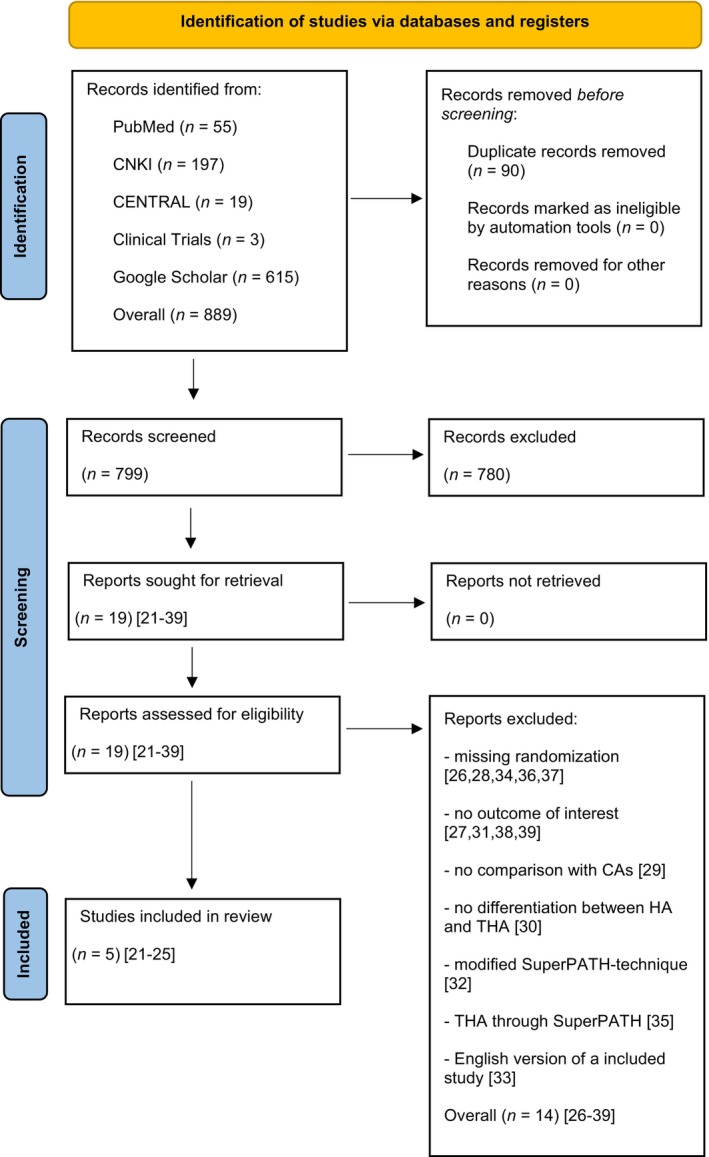
PRISMA flow diagram of the search results and selection according to our inclusion criteria.

The stepwise literature search and selection process identified 19 RCTs^21^, ^22^, ^23^, ^24^, ^25^, ^26^, ^27^, ^28^, ^29^, ^30^, ^31^, ^32^, ^33^, ^34^, ^35^, ^36^, ^37^, ^38^, ^39^ for further consideration through full‐text screening. Of these 19 RCTs, 14 RCTs^26^, ^27^, ^28^, ^29^, ^30^, ^31^, ^32^, ^33^, ^34^, ^35^, ^36^, ^37^, ^38^, ^39^ were excluded (κ = 1.0) for the following reasons: (i) five RCTs^26^, ^28^, ^34^, ^36^, ^37^ were excluded due to missing randomization; (ii) four RCTs reported no outcome of interest;^27^, ^31^, ^38^, ^39^ (iii) one RCT compared two different SuperPATH groups with each other and not with a Cas;^29^ (iv) one RCT did not make any differentiation between HA and THA;^30^ (v) in one RCT a modified SuperPATH technique was used;^32^ (vi) one RCT compared SuperPATH THA with CAs HA;^35^ and (vii) one RCT was an English‐language version of the included study by Jia *et al*.^22^ with a partially identical patient cohort and with fewer outcome parameters.^33^ Finally, a total of 5 RCTs^21^, ^22^, ^23^, ^24^, ^25^ were included in meta‐regression analysis (Figure 1).

### Patient Characteristics

The five included RCTs, involving 404 patients with a mean patient age of 75.68 years (range: 67.15–82.80), were published in Chinese scientific journals between 2017 and 2021. Of these five Chinese RCTs, three had an English abstract^21^, ^22^, ^24^ and two had only a Chinese abstract.^23^, ^25^ Of these 404 patients, 165 (40.84%) were male. The mean BMI of the patients was 25.38 kg/m^2^ (range: 25.13–25.63). The mean preoperative HHS was 30.34 points (range: 15.4–43.16). Almost all patients (98.02%) had a dislocated femoral neck fracture (Garden III or IV) as the indication for surgery. The mean operative time was 72.49 min (range: 48.20–112.96). The mean incision length was 9.79 cm (range: 6.90–17.10). The mean intraoperative blood loss was 165.68 mL (range: 56.24–386.42). The mean time to mobilization was 4.18 days (range: 1.72–8.39). The mean length of hospital stay was 10.67 days (range: 8.40–13.57). Further details of patient and study characteristics are shown in Table 1.

**TABLE 1:** Main characteristics of the RCTs included and patient cohorts.

Author	Year of publication	Origin	Language	English Abstract	Approach	Patients (N)	Dislocated femoral neck fracture (%)	HHS preoperatively (points)	Patient age (years)	Patient age group (years)	Male sex (%)	BMI (kg/m^2^)	Diabetes mellitus (%)	Operation time (min.)	Incision length (cm)	Intraoperative blood loss (ml)	Time to mobilization (days)	Hospitalization period (days)	Complications (N)	Follow‐up period (months)
Dai GH *et al*.^21^	2019	China	Chinese	Yes	S	61	100	33.16	69.90	≤70	36.07	25,63	27.87	71.58	7.06	56.24	1.72	9.16	0	6
					PL	67	100	32.56	70.30	≤70	37.31	25,13	26.87	54.36	12.47	152.47	2.04	13.47	0	6
Jia J *et al*.^22^	2017	China	Chinese	Yes	S	32	100	15.40	77.10	≥71	40.63	NR	28.13	56.00	7.10	132.00	2.60	NR	0	9
					P	32	100	15.60	78.50	≥71	34.38	NR	21.88	52.00	17.10	156.00	5.50	NR	0	9
Wang X and Tian J^23^	2021	China	Chinese	No	S	50	100	43.16	67.84	≤70	52.00	NR	NR	112.96	7.03	193.64	2.73	9.32	0	NR
					PL	50	100	42.16	67.15	≤70	54.00	NR	NR	71.26	9.08	386.42	8.39	13.57	0	NR
Xia LZ *et al*.^24^	2018	China	Chinese	Yes	S	30	83.33	NR	81.00	≥71	26.67	NR	13.33	104.07	7.82	83.00	3.70	NR	0	10
					PL	32	93.75	NR	80.66	≥71	34.38	NR	15.63	89.16	11.84	165.63	6.78	NR	0	10
Zhao L *et al*.^25^	2019	China	Chinese	No	S	25	NR	NR	81.50	≥71	48.00	NR	NR	65.30	6.90	NR	NR	8.40	0	6
					PL	25	NR	NR	82.80	≥71	40.00	NR	NR	48.20	11.50	NR	NR	10.10	2^a^	6

^a^ Deep vein thrombosis.

Abbreviations: BMI, Body Mass Index; HHS, Harris Hip Score; P, posterior; PL, posterolateral; RCT, randomized controlled trial; S, SuperPATH.

### Quality Assessment

**FIGURE 2:**
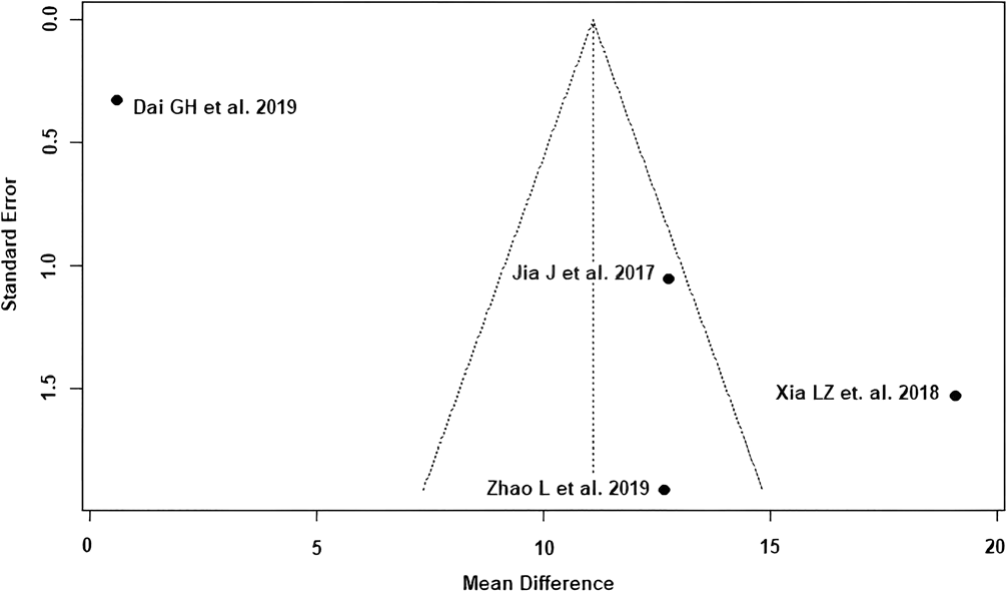
Funnel plot of the HHS ≤1 week postoperatively. The four RCTs^21^, ^22^, ^24^, ^25^ lie within or near the funnel plot triangle, which detects a low risk of publication bias. HHS, Harris hip score.

**FIGURE 3:**
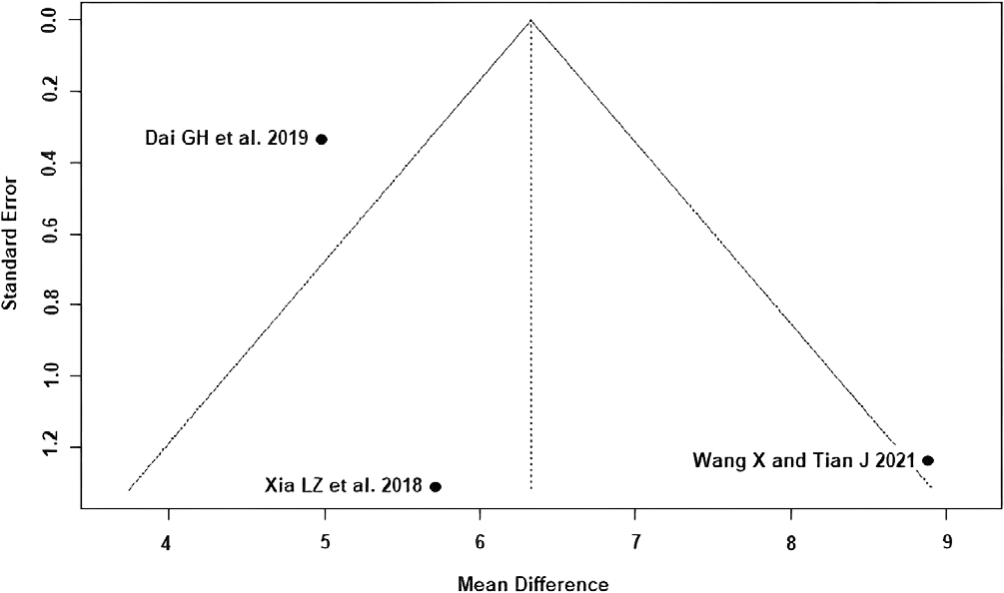
Funnel plot of the HHS 3 months postoperatively. The three RCTs^21^, ^22^, ^24^ lie within or near the funnel plot triangle, which detects a low risk of publication bias. HHS. Harris hip score.

The outcomes HHS ≤1 week postoperatively (Egger's *p*‐value = 0.06) and HHS 3 months postoperatively (Egger's *p*‐value = 0.41) showed a low risk of publication bias (Figures 2 and 3). According to the Cochrane Risk of Bias 2 (RoB 2) tool,^17^ all five included RCTs^21^, ^22^, ^23^, ^24^, ^25^ showed a moderate risk of bias (Table 2). According to the recommendations of the GRADE system,^18^ the outcomes HHS ≤1 week postoperatively and HHS 3 months postoperatively showed low quality of evidence (Table 2).

**TABLE 2:** Risk of bias and level of evidence assessment.

**Risk of bias assessment**							
RCT	Random sequence generation	Allocation concealment	Blinding	Complete outcome data	Selective reporting	Other sources of bias	Overall risk of bias
Dai *et al*.^21^	+	+	?	+	+	+	Moderate RoB
Jia *et al*.^22^	+	+	?	+	+	+	Moderate RoB
Wang and Tian^23^	+	+	?	?	+	+	Moderate RoB
Xia *et al*.^24^	+	+	?	+	+	+	Moderate RoB
Zhao *et al*.^25^	+	+	?	?	+	+	Moderate RoB
**Level of evidence assessment**							
No. of studies	Design	Risk of bias	Inconsistency	Indirectness	Imprecision	Other considerations	Quality of evidence
HHS ≤1 week postoperatively							
4	RCT	Moderate	Serious	No serious indirectness	No serious imprecision	All studies were from China	Moderate
HHS 3 months postoperatively							
3	RCT	Moderate	Serious	No serious indirectness	No serious imprecision	All studies were from China	Moderate

*Note*: Possible results of the RoB assessment: (+): low RoB; (?): unclear, some concerns, or moderate RoB; (−): high RoB. Possible results of the LoE assessments: very low, low, moderate, and high.

Abbreviations: HHS, Harris Hip Score; LoE, Level of Evidence; RCT, randomized controlled trial; RoB, risk of bias.

### Meta‐analysis

**FIGURE 4:**
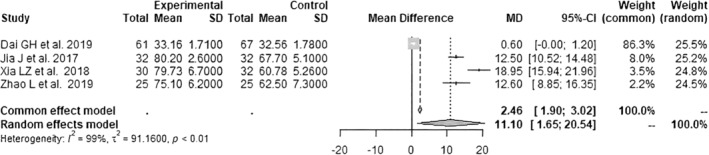
Forest plot of the HHS ≤1 week postoperatively. The MD of the summary measure has positive values, which favors SuperPATH HA (MD = 11.10; 95% CI: 1.65 to 20.54). CI, confidence interval; HHS, Harris hip score; MD, mean difference; SD, standard deviation.

**FIGURE 5:**
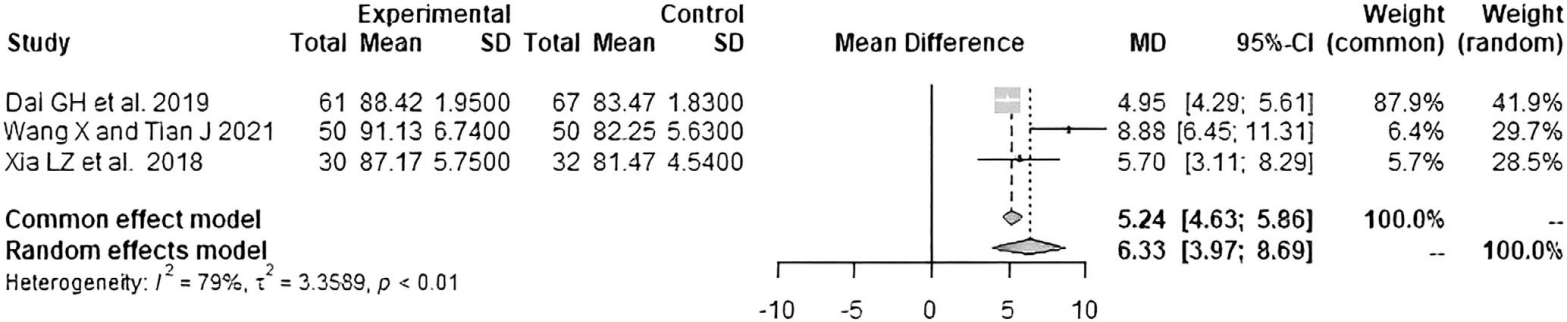
Forest plot of the HHS 3 months postoperatively. The MD of the summary measure has positive values, which favors SuperPATH HA (MD = 6.33; 95% CI: 3.97 to 8.69). CI, confidence interval; HHS, Harris hip score; MD, mean difference; SD, standard deviation.

Data on the HHS ≤1 week postoperatively were pooled from 4 RCTs^21^, ^22^, ^24^, ^25^ with 304 patients (*I* ^2^ = 99%, *p* < 0.01, Figure 4). The SuperPATH group's HHS ≤1 week postoperatively was 11.1 points significantly higher than the CAs group's HHS ≤1 week postoperatively (MD = 11.10; 95% CI: 1.65 to 20.54). Data on the HHS 3 months postoperatively were pooled from 3 RCTs^21^, ^23^, ^24^ with 290 patients (*I* ^2^ = 79%, *p* < 0.01, Figure 5). The SuperPATH group's HHS 3 months postoperatively was 6.3 points significantly higher than the CAs group's HHS 3 months postoperatively (MD = 6.33; 95% CI: 3.97 to 8.69). Weighted mean values or percentages of all predictors and outcome parameters are shown in Table 3.

**TABLE 3:** Weighted mean values or percentages of all predictors and outcome parameters.

Patient‐related predictor	SuperPATH	CA	Overall
Dislocated femoral neck fracture (%)	98.34	98.90	99.35
HHS preoperatively (mean; range in points)	28.06; 15.40–43.16	27.74; 15.60–42.16	27.90; 15.40–43.16
Patient age (mean; range in years)	73.87; 67.84–81.50	74.06; 67.15–82.80	73.97; 67.15–82.8
Male sex (%)	46.95	48.05	47.51
Diabetes mellitus (%)	23.08	22.60	22.83
Operation time (mean; range in min)	86.55; 56.00–112.96	63.81; 48.20–89.16	75.06; 48.20–112.96
Incision length (mean; range in cm)	6.89; 6.90–7.82	11.19; 9.08–17.1	9.06; 6.90–17.10
Intraoperative blood loss (mean; range in mL)	143.80; 56.24–193.64	247.97; 152.47–386.42	196.47; 56.24–386.42
Time to mobilization (mean; range in days)	2.69; 1.72–3.70	6.40; 2.04–8.39	4.57; 1.72–8.39
Hospitalization time (mean; range in days)	9.06; 8.40–9.32	13.28; 10.10–13.57	11.19; 8.40–13.57
Outcome parameter	SuperPATH	CA	Overall
HHS ≤1 week postoperatively (mean; range in points)	59.86; 33.16–80.20	50.36; 32.56–67.70	54.98; 32.56–80.20
HHS 3 months postoperatively (mean; range in points)	89.12; 87.17–91.13	82.63; 81.47–83.47	85.78; 81.47–91.13

Abbreviations: CA, conventional approach; HHS, Harris Hip Score.

### Meta‐regression Analysis

#### Patient‐related Predictors of HHS ≤1 Week Postoperatively

**FIGURE 6:**
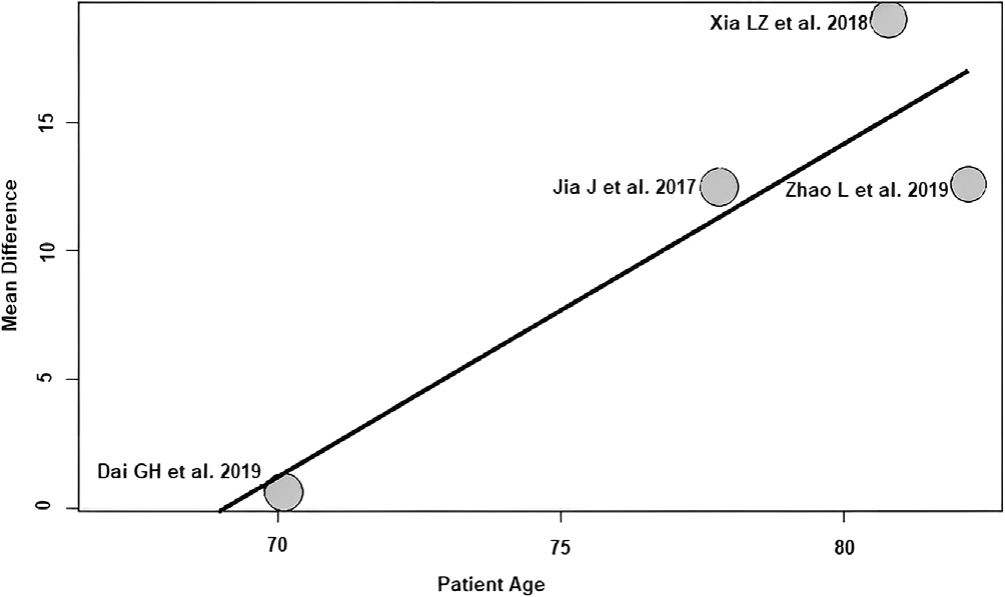
Bubble plot of the predictor patient age and the outcome parameter HHS ≤1 week postoperatively. The slope of the regression line is large (steep line), which shows that the predictor has a strong influence on the outcome variable. HHS, Harris hip score.

Patient age: of the four RCTs^21^, ^22^, ^24^, ^25^ with 304 patients reporting patient age demographics, a positive association (predictor estimate = 1.29) was seen between patient age and HHS ≤1 week postoperatively. With the increase of the average patient age by 1 year, the effect size for HHS ≤1 week postoperatively increased on average by 1.29 points (*p* < 0.01; Figure 6).

**FIGURE 7:**
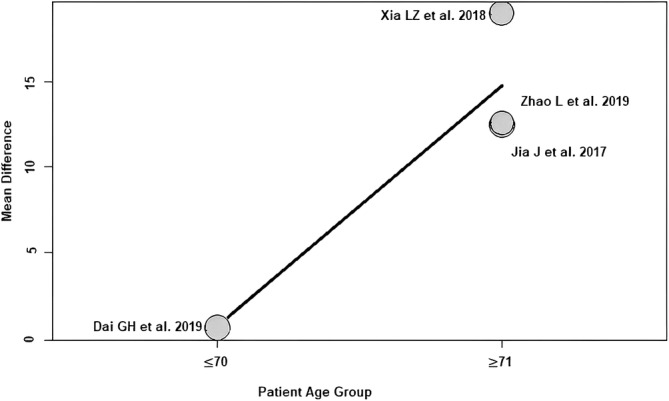
Bubble plot of the predictor patient age group and the outcome parameter HHS ≤1 week postoperatively. The slope of the regression line is large (steep line), which shows that the predictor has a strong influence on the outcome variable. HHS, Harris hip score.

Patient age group: of the four RCTs^21^, ^22^, ^24^, ^25^ with 304 patients reporting patient age demographics and grouped in age groups, a positive association (predictor estimate = 14.07) was seen between patient age group and HHS ≤1 week postoperatively. When the mean age of the patients was at least 71 years, the effect size for HHS ≤1 week postoperatively increased by an average of 14.07 points (*p* < 0.01; Figure 7) compared with RCTs with younger patients (mean age ≤70 years).

**FIGURE 8:**
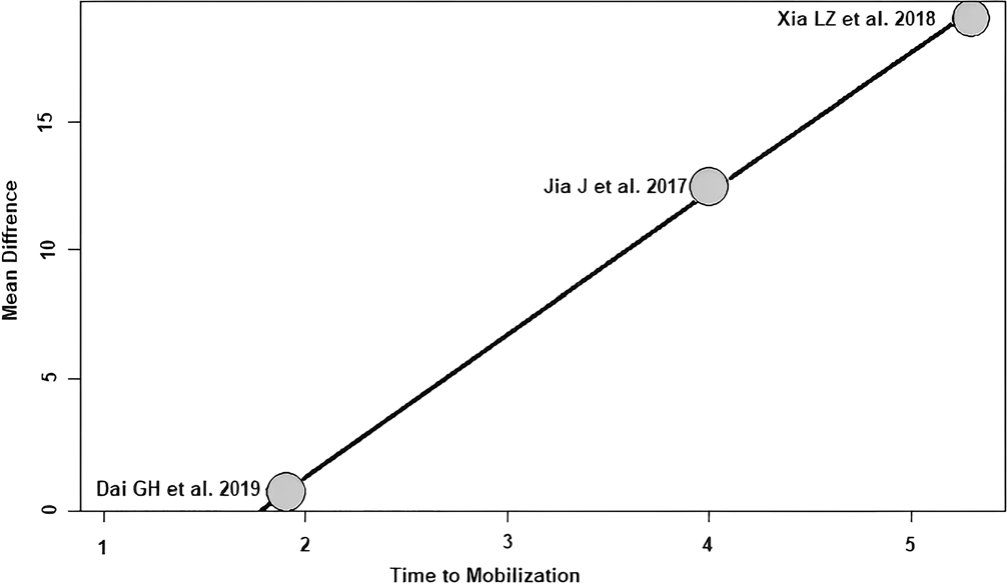
Bubble plot of the predictor time to mobilization and the outcome parameter HHS ≤1 week postoperatively. The slope of the regression line is large (steep line), which shows that the predictor has a strong influence on the outcome variable. HHS, Harris hip score.

Time to mobilization: of the three RCTs^21^, ^22^, ^24^ with 254 patients reporting the time to mobilization, a positive association (predictor estimate = 5.51) was seen between the time to mobilization and HHS ≤1 week postoperatively. With the increase of the average time to mobilization by 1 day, the effect size for HHS ≤1 week postoperatively increased on average by 5.51 points (*p* < 0.01; Figure 8).

The remaining predictors (dislocated femoral neck fracture, operation time, incision length, intraoperative blood loss, male sex, and diabetes mellitus) were statistically insignificant (Table 4).

**TABLE 4:** All results of the meta‐regression analysis for all patient‐related predictors and all outcome parameters.

Outcome	Predictor	Number of RCTs	Number of patients	*I* ^2^	*𝜏* ^2^	QE.p.val	Predictor.estimate	Predictor.p.val
**HHS ≤1 week postoperatively**	Dislocated femoral neck fracture	3	254	99.21	70.13	<0.01***	−1.10	0.23
	HHS preoperatively	2	192	‐	‐	‐	‐	‐
	Patient age	4	304	90.36	16.61	<0.01**	1.29	<0.01**
	Patient age group	4	304	86.05	12.97	0.01**	14.07	<0.01***
	Operation time	4	304	98.71	61.50	<0.01***	0.21	0.37
	Incision length	4	304	98.47	85.73	<0.01***	0.44	0.92
	Intraoperative blood loss	3	254	98.23	97.93	<0.01***	0.32	0.39
	Time to mobilization	3	254	13.93	0.21	0.68	5.51	<0.01***
	Hospitalization period	2	178	‐	‐	‐	‐	‐
	Male sex	4	304	99.06	79.33	<0.01***	−0.40	0.67
	Diabetes mellitus	3	254	98.43	42.89	<0.01***	−1.19	0.09
**HHS 3 months postoperatively**	Dislocated femoral neck fracture	3	290	87.90	6.01	<0.01**	0.09	0.76
	HHS preoperatively	2	228	‐	‐	‐	‐	‐
	Patient age	3	290	88.22	5.23	<0.01**	−0.14	0.60
	Patient age group	3	290	87.90	6.01	<0.01**	−1.01	0.76
	Operation time	3	290	70.56	3.88	0.05	0.07	0.42
	Incision length	3	290	18.65	0.21	0.58	−2.12	<0.01**
	Intraoperative blood loss	3	290	4.48	0.05	0.80	0.02	<0.01**
	Time to mobilization	3	290	63.14	2.82	0.10	0.71	0.27
	Hospitalization period	2	228	‐	‐	‐	‐	‐
	Male sex	3	290	64.52	1.31	0.13	0.17	0.09
	Diabetes mellitus	2	190	‐	‐	‐	‐	‐

*Notes*: A meta‐regression analysis was impossible for predictors: “HHS preoperatively” and “hospitalization period.” *Statistically significant.

Abbreviations: HHS, Harris hip score; RCT, randomized controlled trial.

*statistically significant

** high statistically significant

*** very high statistically significant.

#### Patient‐related Predictors of HHS 3 Months Postoperatively

**FIGURE 9:**
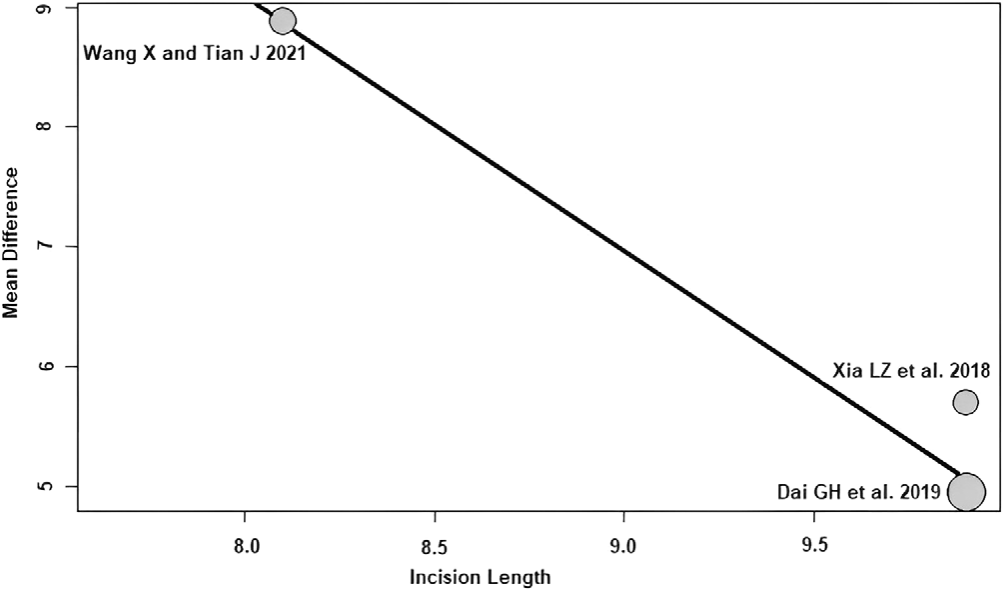
Bubble plot of the predictor incision length and the outcome parameter HHS 3 months postoperatively. The slope of the regression line is large (steep line), which shows that the predictor has a strong influence on the outcome variable. HHS, Harris hip score.

Incision length: of the three RCTs^21^, ^23^, ^24^ with 290 patients reporting incision length, a negative association (predictor estimate = −2.12) was seen between incision length and HHS 3 months postoperatively. With the increase of the average incision length by 1 cm, the effect size for HHS 3 months postoperatively decreased on average by 2.12 points (*p* < 0.01; Figure 9).

**FIGURE 10:**
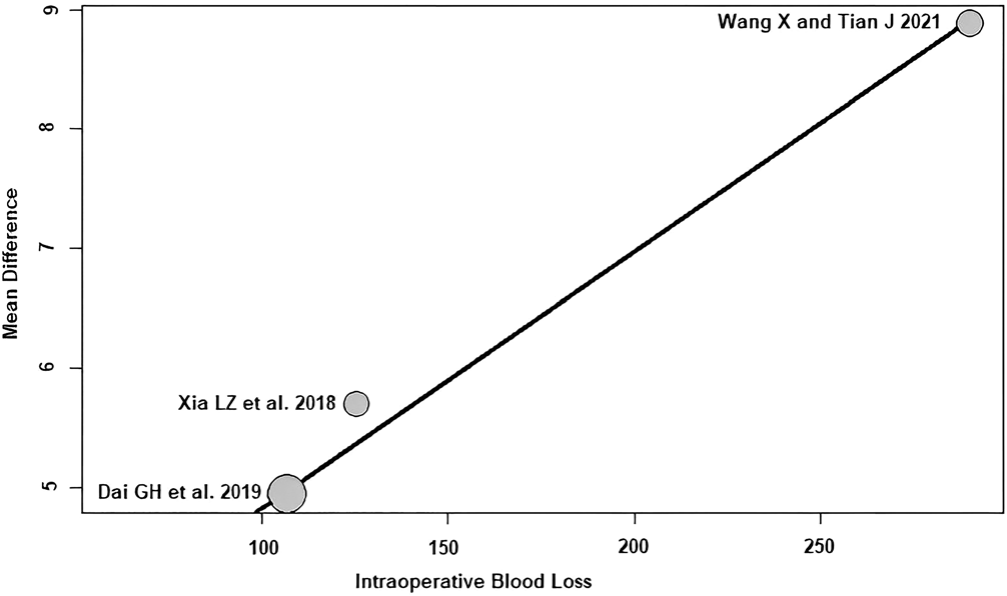
Bubble plot of the predictor intraoperative blood loss and the outcome parameter HHS 3 months postoperatively. The slope of the regression line is large (steep line), which shows that the predictor has a strong influence on the outcome variable. HHS, Harris hip score.

Intraoperative blood loss: of the three RCTs^21^, ^23^, ^24^ with 290 patients reporting the intraoperative blood loss, a positive association (predictor estimate = 0.02) was seen between intraoperative blood loss and HHS 3 months postoperatively. With the increase of the average intraoperative blood loss by 1 mL, the effect size for HHS 3 months postoperatively increased on average by 0.02 points (*p* < 0.01; Figure 10).

The remaining predictors (dislocated femoral neck fracture, age, age group, operation time, incision length, intraoperative blood loss, time to mobilization, male sex, and diabetes mellitus) were statistically insignificant (Table 4).

## Discussion

This meta‐regression analysis, including five RCTs with 404 patients, identified patient age and time to mobilization as predictors of the effect size of the HHS ≤1 week postoperatively, and incision length and intraoperative blood loss as predictors of the effect size of the HHS 3 months postoperatively. The value of this study stems from the use of high‐quality statistical methods and the inclusion of only RCTs. Furthermore, it is the first study of its kind to investigate the patient‐related predictors influencing the superior functional outcome of SuperPATH HA compared to CA HA.

### Patient‐related Predictors of HHS ≤1 Week Postoperatively

The outcomes parameter HHS ≤1 week postoperatively and HHS 3 months postoperatively showed a low risk of publication bias and a low quality of evidence. All of the included RCTs had a moderate risk of bias. SuperPATH HA had 11.1 points and 6.3 points higher HHS ≤1 week postoperatively and 3 months postoperatively than CA HA. When investigating significant differences in functional outcome after hip surgery, the question of clinical relevance always arises. The minimal clinically significant difference (MCID) in hip function after surgery has been reported to be no less than 7.9 points,^40^ as measured by the HHS. This means that the difference in the HHS ≤1 week postoperatively between SuperPATH HA and CA HA is highly clinically relevant. A total of nine potential patient‐related predictors were considered: dislocated femoral neck fracture, patient age, patient age group, operation time, incision length, intraoperative blood loss, time to mobilization, male sex, and diabetes mellitus.

This meta‐regression analysis showed a positive association between patient age and the effect size of HHS ≤1 week postoperatively. The effect size of HHS ≤1 week postoperatively increased by an average of 1.29 points for each 1‐year increase in mean age. In other words, the difference found in the meta‐analysis between SuperPATH HA and CA HA changes by 1.29 HHS points per patient‐year in favor of SuperPATH. In addition to the patient age predictor examined, patients were divided into an elderly (≤70 years) and younger (≥71 years) patient age groups according to their age. A positive association was found between patient age groups and the effect size of HHS ≤1 week postoperatively. When the mean age of the patients was at least 71 years, the effect size for HHS ≤1 week postoperatively increased by an average of 14.07 points compared with RCTs with younger patients (mean age ≤70 years). Elderly patients appear to benefit more from SuperPATH HA than from CA HA compared with younger patients. This finding is consistent with the results of a recent meta‐regression analysis^13^ investigating predictors of minimally invasive THA outcome and CA THA outcome. This study^13^ also found a strong positive association between patient age and the effect size of HHS ≤3 months postoperatively. A reasonable explanation for the similar findings is certainly the obvious fact that younger patients are better able to compensate for tissue trauma than elderly patients. This finding is very important because in daily hip surgery practice we still tend to limit less traumatic minimally invasive techniques to elective THA patients, while elderly trauma patients, who would benefit from less soft tissue trauma, are still mostly operated on through CAs. A 2018 analysis of a Medicare database of 233,984 patients confirms this. The authors Fan *et al*. showed that minimally invasive surgery has advantages over conventional surgery in terms of fewer complications, shorter hospital stays, lower readmission and mortality rates, and lower healthcare costs.^41^ In addition, this analysis found that minimally invasive surgery is underused in the population that could benefit most from it.^41^ In particular, elderly patients were offered minimally invasive surgery rather than conventional surgery at a lower rate than the general population.^41^

Furthermore, a positive association between time to mobilization and the effect size of HHS ≤1 week postoperatively was found. The effect size of HHS ≤1 week postoperatively increased by an average of 5.51 points for each 1‐day increase in mean time to mobilization. In other words, the difference found in the meta‐analysis between SuperPATH HA and CA HA changes by 5.51 HHS points per day of time to mobilization in favor of SuperPATH. This association seems obvious and is consistent with the findings in the specialist literature.^42^

### Patient‐related Predictors of HHS 3 Months Postoperatively

This meta‐regression analysis showed a negative association between incision length and the effect size of HHS 3 months postoperatively. The effect size of HHS 3 months postoperatively decreased by an average of 2.12 points for each 1 cm increase in mean incision length. In other words, the difference found in the meta‐analysis between SuperPATH HA and CA HA changes by 2.12 HHS points per 1 cm increase in incision length to the disadvantage of SuperPATH. In our patient cohort, the mean incision length for SuperPATH HA was 6.89 cm, ranging from 6.90 to 7.82 cm, and the mean incision length for CA HA was 11.19 cm, ranging from 9.08 to 17.1 cm. This finding is consistent with the recent meta‐regression analysis^13^ investigating predictors of outcome for minimally invasive THA and CA THA. There, the effect size of HHS ≥6 months postoperatively decreased by an average of 0.82 points for each 1 cm increase in mean incision length.^13^

Furthermore, a positive association between intraoperative blood loss and the effect size of HHS 3 months postoperatively was found. The effect size of HHS 3 months postoperatively increased by an average of 0.02 points for each 1 mL increase in mean intraoperative blood loss. In other words, the difference found in the meta‐analysis between SuperPATH HA and CA HA changes by 0.02 HHS points per 1 mL of intraoperative blood loss in favor of SuperPATH. Although the results are statistically significant, the HHS difference of 0.02 points is certainly not clinically relevant. Even if the intraoperative blood loss were 100 times greater, the resulting HHS difference of 2 points would not meet the MCID value of 7.9 points.^40^

### Comparison with the Literature

To begin the comparison with recent literature, the first meta‐analysis investigating SuperPATH HA and CA HA outcomes^12^ was recently published. This meta‐analysis, which included nine RCTs and 762 patients, showed that SuperPATH leads to a better early functional outcome and reduced incision length, intraoperative blood loss, postoperative drainage volume, time to mobilization, postoperative pain score, and length of hospital stay.^12^ The superior performance of SuperPATH HA raises the question of the factors influencing it, which the present meta‐regression analysis attempts to answer.

In a further comparison with the literature, there are some related studies that provide interesting results worth mentioning. A 2022 systematic review of 9486 THAs investigated possible factors that may influence the surgical outcome of minimally invasive THA. It found that older age and higher BMI may be negative prognostic factors for minimally invasive THA and that the clinical outcome is strongly influenced by patients' preoperative status.^43^ A 2019 retrospective study of 116 patients found that return to work after hip and knee arthroplasty was not significantly influenced by the type of surgery or the physical demands of the job. Patients returned to work between 5.9 and 7.7 weeks after hip/knee replacement. Rehabilitation, desire, and need facilitated return to work, while pain, fatigue, and medical restrictions hindered return to work.^44^ A 2010 study of 43 female patients with unilateral hip osteoarthritis found that the stage of osteoarthritis and changes in leg length discrepancies were the factors that most influenced gait improvement after total hip arthroplasty.^45^ A 2015 systematic review of 37 articles found a strong association between postoperative pain after knee or hip arthroplasty and the following preoperative factors: female gender, low socioeconomic status, higher pain, comorbidities, low back pain, poor functional status, and psychological factors (depression, anxiety, or catastrophic pain).^46^

### Strengths and Limitations

The following strengths and limitations were identified: (i) this meta‐regression analysis examines which factors influence the effect size in the outcome between the SuperPATH HA and CA HA; it does not simply examine which factors influence HA; (ii) a wide range of patient‐related predictors were examined using meta‐regression analysis based on random‐effects meta‐analysis using the Hartung–Knapp–Sidik–Jonkman method; (iii) the total size of five RCTs with 404 patients is a moderate number for this type of research; (iv) the medium‐term and long‐term HA outcomes were not considered; (v) complications after HA are an important outcome that could not be studied due to insufficient data; and (vi) in some cases, information on standard deviation was not reported, and it was inserted via imputation.

## Conclusion

Patient age, time to mobilization, incision length, and intraoperative blood loss were identified as predictors of the effect size of early postoperative functional outcome as measured by HHS. Elderly patients, particularly those over 70 years of age, appear to benefit from SuperPATH HA. Based on these findings, and taking into account our limitations, we recommend that the use of minimally invasive SuperPATH HA in elderly patients should be more widely considered and not limited to elective THA patients.

## Author Contributions

NR and MV performed the systematic literature search and data extraction. RH, NR, and RP carried out all statistical calculations. NR drafted the manuscript. HTH and MS assisted in the preparation and design of tables and figures. DD and RB supervised the entire work. The authors read and approved the final version of the manuscript.

## Conflict of Interest Statement

No conflict of interest.

## Ethics Statement

As this is a systematic review and meta‐regression analysis, an ethical statement is not applicable.

## Supporting information


**Data S1.** Supporting Information.
